# Contrast-Induced Encephalopathy After Simultaneous Embolization of Multiple Intracranial Aneurysms: A Case Report and Management

**DOI:** 10.7759/cureus.103523

**Published:** 2026-02-13

**Authors:** Hilmiye Tokmak, Ferhat Gelmez, Ahmet Yabalak, Muhammed Nur ÖĞün

**Affiliations:** 1 Department of Neurology, Niğde Eğitim ve Araştırma Hastanesi, Niğde, TUR; 2 Department of Neurology, Faculty of Medicine, Düzce University, Düzce, TUR; 3 Department of Neurology, Bolu Abant İzzet Baysal University, Bolu, TUR

**Keywords:** cerebral edema, contrast-induced encephalopathy, endovascular treatment, intracranial aneurysm, iodinated contrast media

## Abstract

Contrast-induced encephalopathy (CIE) is a rare but potentially serious complication associated with the administration of contrast media. We report the case of a 70-year-old woman undergoing diagnostic cerebral angiography for asymptomatic common carotid artery stenosis who developed transient drowsiness during the procedure. Shortly after angiography, she experienced generalized tonic-clonic seizures involving the left upper and lower extremities, accompanied by leftward gaze deviation. Computed tomography revealed cortical high-density signals consistent with contrast leakage, leading to a diagnosis of CIE. This case highlights the need for vigilant monitoring for acute neurological changes during and immediately after angiographic procedures, even when only small amounts of contrast agents are used.

## Introduction

Contrast-induced encephalopathy (CIE) is a rare but potentially serious neurological complication associated with the administration of iodinated contrast media [1,2]. It is characterized by a broad spectrum of transient neurological symptoms, including altered mental status, seizures, focal neurological deficits, and cortical blindness [2,3]. Although most frequently reported after cerebral angiography and endovascular procedures, CIE has also been described following contrast-enhanced computed tomography, coronary angiography, and peripheral vascular interventions [1,2]. Because its clinical presentation may closely mimic acute ischemic stroke or intracranial hemorrhage, early recognition is essential to avoid unnecessary interventions and delays in appropriate management [3].

The exact pathophysiology of CIE remains incompletely understood. Proposed mechanisms include transient disruption of the blood-brain barrier and the direct neurotoxic effects of contrast agents, leading to vasogenic cerebral edema [3-5]. Both ionic and non-ionic, hyperosmolar and low-osmolar contrast media have been implicated. Although the overall incidence of CIE is low, ranging from 0.3% to 1%, the risk increases in neurointerventional procedures that require large volumes of contrast, particularly during cerebral aneurysm embolization [2,4]. Reported risk factors include high contrast dose, posterior circulation interventions, impaired renal function, hypertension, and diabetes mellitus [2,5].

Most cases of CIE demonstrate a benign and reversible course with complete clinical recovery within 48-72 hours under supportive treatment [2,6,7]. However, persistent neurological deficits and fatal outcomes have been reported, underscoring the clinical relevance of this entity [4]. Awareness of CIE is therefore critical for clinicians involved in contrast-based diagnostic and therapeutic procedures.

In this report, we present a case of CIE that developed after simultaneous stent-assisted coil embolization of two unruptured internal carotid artery aneurysms. This case highlights the potential role of increased regional contrast load and impaired renal function in the development of CIE and emphasizes the importance of early recognition and prompt supportive management.

## Case presentation

A 70-year-old woman with a medical history of cholecystectomy, knee arthroscopy, hyperthyroidism, and nephrolithiasis was found to have two unruptured saccular aneurysms of the left internal carotid artery, measuring 5.9 × 5.3 × 6.3 mm proximally and 8.5 × 8.3 × 6.2 mm distally. The aneurysms were incidentally detected on CT angiography performed for cerebrovascular evaluation, with no history of subarachnoid hemorrhage, thunderclap headache, or acute neurological presentation.

Pre-procedural laboratory evaluation revealed a serum creatinine level of 1.18 mg/dL, urea of 67 mg/dL, and an estimated glomerular filtration rate of 42 mL/min/1.73 m², indicating moderate renal impairment. Given the increased risk of contrast-related complications, nephroprotective measures, including intravenous sodium bicarbonate infusion and N-acetylcysteine administration, were initiated 24 hours prior to the procedure (Table 1).

**Table 1:** Pre-procedural laboratory findings

Parameter	Patient Value	Reference Range	Unit
Creatinine	1.18	0.6 – 1.2	mg/dL
Urea	67	10 – 50	mg/dL
Estimated GFR	42	> 60	mL/min/1.73 m²

**Figure 1:**
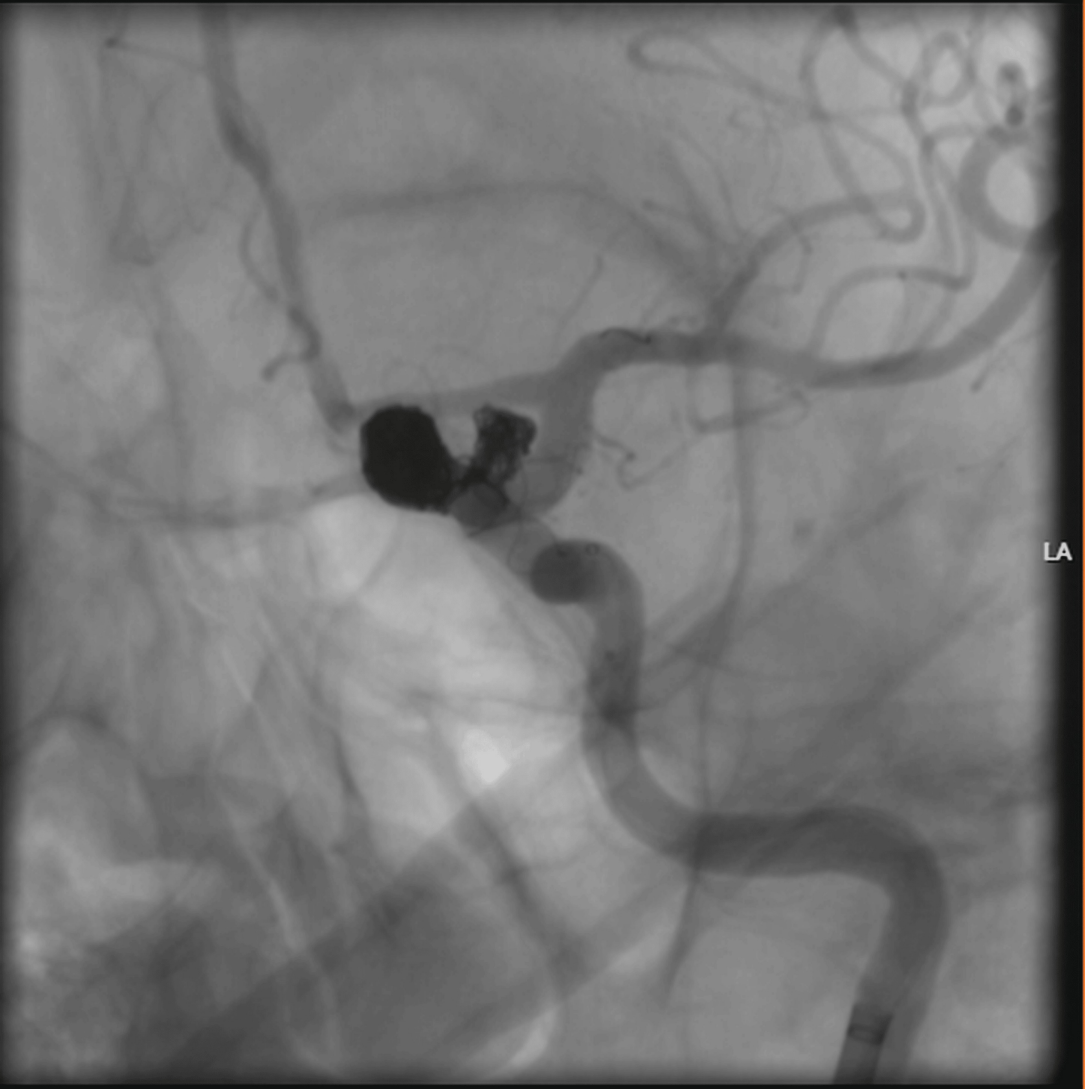
Aneurysm coil embolization

**Figure 2:**
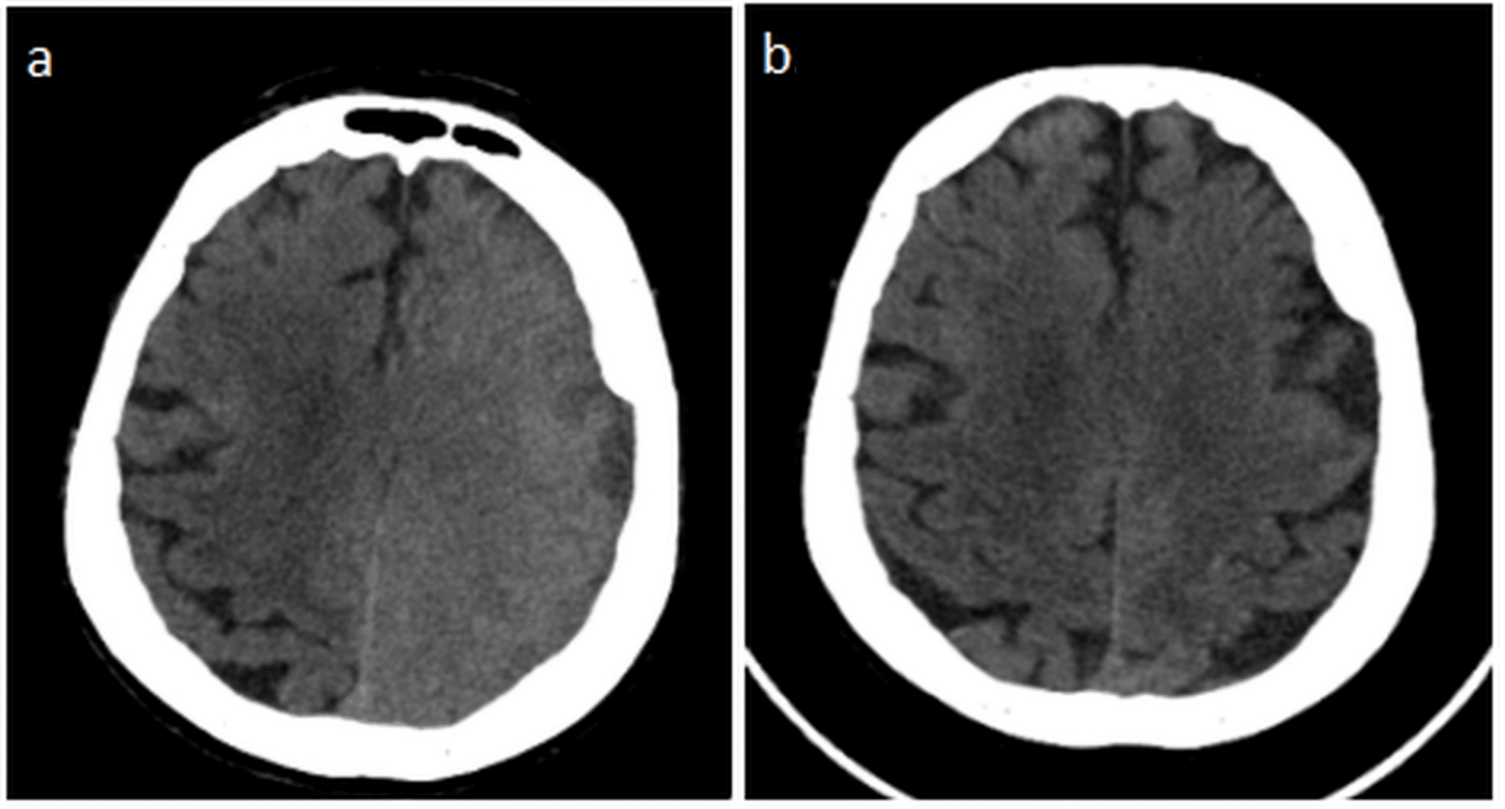
(a) Early post-procedure non-contrast brain CT demonstrating left hemispheric cortical edema and loss of gray–white matter differentiation, with no hemorrhage or mass effect, compatible with contrast-induced encephalopathy. (b) Follow-up CT after supportive therapy showing complete resolution of edema and normalization of cortical appearance

Under general anesthesia, both left internal carotid artery aneurysms were treated in a single session using stent-assisted coil embolization. The procedure was technically complex and lasted approximately four hours, during which a total of 250 mL of iohexol (300 mg I/mL) was administered, corresponding to an estimated total iodine load of approximately 62.5 g (Figure 1). After awakening from anesthesia, the patient developed agitation, delirium, and right-sided mild hemiparesis, prompting urgent brain CT imaging (Figure 2a). The CT scan demonstrated cerebral edema with loss of gray-white matter differentiation in the left cerebral hemisphere. The patient subsequently underwent diagnostic angiography. As the proximal and distal cerebral vessels were patent and no contrast extravasation was observed, a diagnosis of CIE was considered. Following an initial dose of 16 mg intravenous dexamethasone, maintenance therapy with dexamethasone (4×4 mg), levetiracetam (2×500 mg), and 3% hypertonic NaCl solution (4×100 mL) was initiated.

At the 48th hour after the procedure, agitation had markedly improved, although mild sensory aphasia persisted. By the 72nd hour, the neurological examination had completely returned to normal. Follow-up CT imaging demonstrated complete resolution of the edema (Figure 2b). Dexamethasone and hydration therapy were gradually discontinued. The neurological examination at the three-month follow-up was also normal.

## Discussion

CIE is a rare complication of diagnostic angiography and percutaneous interventions. Although it is most commonly reported after cerebral angiography, it has also been described following procedures such as contrast-enhanced CT, cardiac angiography, and peripheral angiography [1]. Clinically, it can mimic stroke due to the presence of encephalopathy, seizures, cortical blindness, and focal neurological deficits, and radiologically, it may resemble subarachnoid hemorrhage [2].

Although the pathophysiology has not been fully elucidated, temporary disruption of the blood-brain barrier and the direct neurotoxic effect of contrast media are considered responsible. Increased permeability of the barrier in the occipital cortex is thought to explain why visual symptoms predominate in some cases [3].

CIE has been associated with various contrast agents, including ionic or non-ionic, hyperosmolar or low-osmolar formulations [4]. While its overall incidence has been reported between 0.3 and 1%, this rate may increase up to 2.9% in cerebral aneurysm coiling procedures where high volumes of contrast are used [5]. According to a study conducted by Li et al., high-dose contrast administration, posterior circulation procedures, impaired renal function, hypertension, and diabetes are prominent risk factors [8].

Brain CT is the first-line imaging modality in diagnosis. Loss of gray-white matter differentiation, sulcal effacement, and vasogenic edema are typical findings. However, preservation of distal vessel patency and the absence of extravasation are critically important in differentiating CIE from ischemic and hemorrhagic events [6]. In our patient, the presence of these radiological features enabled a rapid diagnosis.

Although there is no specific treatment for CIE, supportive approaches such as hydration, anti-edema therapy, steroids, and seizure prophylaxis are used [7]. Most of the literature reports that clinical manifestations resolve completely within 48-72 hours with supportive treatment; similarly, in our case, the neurological status returned entirely to normal within 72 hours. These approaches are also consistently described in published case reports and review articles.

CIE is an uncommon but clinically significant complication that can mimic acute cerebrovascular events. In this case, the rapid onset of symptoms following contrast exposure, characteristic CT findings, preserved vessel patency, and complete recovery strongly supported the diagnosis of CIE. Alternative diagnoses such as acute ischemic stroke, intracranial hemorrhage, thromboembolic complications, posterior reversible encephalopathy syndrome, and metabolic or anesthesia-related encephalopathy were considered and excluded based on clinical course and imaging findings. Compared with previously reported cases, our patient developed CIE after the simultaneous treatment of two ICA aneurysms within the same vascular territory, resulting in a relatively high regional contrast burden in a single session. In addition, moderate renal impairment may have further reduced contrast clearance. These combined factors distinguish our case from typical single-lesion interventions and support the concept that cumulative regional contrast exposure is an important contributor to CIE risk. The rapid radiological reversibility on follow-up imaging further argued against acute ischemic stroke.

In our case, performing interventions on two ICA aneurysms within the same arterial supply area, thereby increasing the regional contrast load, was considered a significant trigger for the development of CIE. Additionally, it has been reported in the literature that impaired renal function increases susceptibility to central nervous system toxicity by affecting contrast clearance. From this perspective, the comorbidities present in our patient may have predisposed her to the development of CIE. Most cases reported in the literature have developed CIE after posterior circulation interventions, while cases reported following anterior circulation aneurysm treatment are more limited. Nevertheless, it is noteworthy that in our patient, unlike the transient cortical blindness more frequently described in posteriorly located cases, focal neurological deficits were predominant. Furthermore, the complete resolution of clinical and radiological findings within 72 hours, similar to many previously reported cases, once again demonstrates the reversible nature of CIE with supportive treatment.

## Conclusions

This case demonstrates that CIE, although rare and typically reversible, may occur following complex endovascular procedures involving high contrast volumes, especially in patients with renal impairment. Early recognition and supportive management are essential for favorable outcomes. Future studies are needed to better define safe contrast thresholds and risk-reduction strategies in high-risk patients.
